# Oil foam based on dairy proteins particles and surfactant

**DOI:** 10.1016/j.crfs.2026.101343

**Published:** 2026-02-07

**Authors:** Luisa Azevedo Scudeller, Annika Feichtinger, Séverine Bellayer, Thierry Six, Manon Hiolle, Guillaume Delaplace, Elke Sholten, Anne-Laure Fameau

**Affiliations:** a INRAE, University Lille, CNRS, Centrale Lille, UMET, Lille, 59000, France; b Physics and Physical Chemistry of Foods, Wageningen University and Research, Bornse Weilanden 9, Wageningen, 6708 WG, the Netherlands; c Ingredia – Ingredia Dairy Experts, Arras, 62033, France

**Keywords:** Oil foam, Surfactant, Dairy protein, Edible particles, Casein, Whey protein

## Abstract

Fat plays a crucial role in food applications, affecting texture, mouthfeel, and stability. However, traditional solid fats are rich in saturated fats, which pose health risks. To address this, the food industry is exploring alternatives such as replacing saturated fats with unsaturated fats or lowering overall fat content. One promising approach is the use of aerated oils or oil foams, where air is incorporated into oil to reduce fat while imparting solid-like properties.

This study aimed to investigate the potential of different dairy proteins to stabilize oil foams in addition to using food-grade surfactants as stabilizers. Protein powders based on casein micelles or whey proteins, with or without milk fat and lactose, were tested alongside a model surfactant to evaluate their effects on sunflower oil foam formation and stability. The impact of protein type, concentration, oil type, and temperature on foam properties was systematically assessed.

Foam stability was mainly influenced by the protein powders’ tendency to sediment and/or the presence of agglomerates rather than their protein content or type. Powders high in milk fat and lactose were denser, contained less occluded air, and produced unstable foams, while fat- and lactose-free powders, especially those rich in casein, formed highly stable foams. Adding dairy powders rich in casein at 30 wt% significantly improved long term foam stability at room temperature. These findings demonstrate that proteins powders can improve oil foam stability by reducing drainage depending on the powder composition. Such protein-stabilized oil foams offer potential to improve the nutritional profile of diverse food products.

## Highlights

• Oil foam stability was successfully improved with dairy powder alongside surfactants.
• A higher dairy powder concentration leads to higher oil foam stability.
• Faster sedimentation of the powder in the oil phase led to foam destabilization.
• The occluded air amount is the key parameter governing the powder sedimentation.
• Fat- and lactose-free casein powders produced the most stable foams.

## Graphical abstract

Created in BioRender. Fameau, A. (2025) https://BioRender.com/x53d216.

**Figure:**
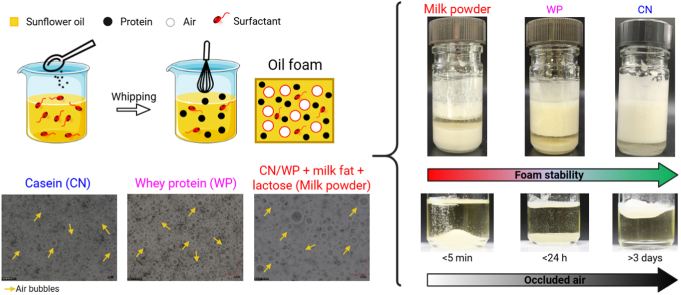

## 1 Introduction

Foams are dispersions of gas bubbles in a liquid phase that are widely applied in food, pharmaceutical and cosmetic products (Binks and Vishal, 2021; Fameau and Binks, 2021). In the food industry, there is a growing emphasis on developing clean labels, as well as enhancing nutritional value and the shelf life to reduce food waste. For example, the food industry aims to replace saturated fat by unsaturated fat and to lower the fat content of the final product (Patel and Dewettinck, 2016). However, this replacement is not simple, since it leads to a loss of texture, structure, and mouthfeel provided by solid fats. A recent approach is to use oil foams or oleofoams (Chisholm et al., 2021). Oil foams consist of an edible liquid oil as the continuous phase and a dispersed gas phase, stabilized by foaming agents (adsorbed solid particles or molecular surfactants) (Fameau and Binks, 2021; Fameau and Saint-Jalmes, 2017; Grossi et al., 2024a; Heymans et al., 2017). Oil foams are reduced in overall fat content and, compared to oil alone, enhanced in solid-like properties, due to the incorporation of air bubbles. The presence of air bubbles can also provide new appealing textures and sensorial properties. In addition, due to the absence of water, the microbial spoilage is reduced ensuring a very long-term stability even above room temperature (Grossi et al., 2024b). Moreover, recent results on the oxidative stability of oleogels before and after foaming showed similar trends, suggesting that incorporating air into this type of matrix does not significantly impair oxidative stability (Ribourg-Birault et al., 2024).

However, producing oil-based foams differs significantly from the process used to create aqueous foams. Traditional hydrocarbon-based surfactants such as sodium dodecyl sulfate, commonly employed in aqueous foams, cannot be used in oil foam formation, because their adsorption at the oil-air interface is energetically unfavorable due to their lower surface activity (Fameau and Saint-Jalmes, 2017). This makes oil-based foams more challenging to produce than aqueous foams. Oil foam formation described in the literature is mainly based on the use of crystalline particles from fatty components (Fameau and Saint-Jalmes, 2017). Often, high melting triglycerides (TAGs) (Binks and Marinopoulos, 2017; Goibier et al., 2019; Liu and Binks, 2021a; Mishima et al., 2016; Mishra et al., 2020) or lipid surfactants, such as mono/or diglycerides (Brun et al., 2015; Gunes et al., 2017; Heymans et al., 2018; Lei et al., 2020; Melchior et al., 2024; Saremnejad et al., 2020; Shrestha et al., 2006) are used. In addition, mixtures of different components have also been studied: long chain fatty alcohols and/or fatty acids (Callau et al., 2020), monoglycerides and phytosterols (Truong et al., 2019) and sucrose ester and lecithin (Karp, 2016). The oil foams are efficiently stabilized by the presence of crystals surrounding the air bubbles and in high amount in the continuous liquid phase, thereby preventing both oil drainage and bubble coalescence (Liu and Binks, 2021b; Qiu et al., 2022; Tirgarian and Farmani, 2023). Recently, also three surfactant families have been shown to lead to oil foams, due to their surface activity at the air/oil surface; sorbitan ester (Liu and Binks, 2022), sucrose ester (Liu and Binks, 2021b), and citric acid ester (Ugarte-Pereyra et al., 2025). The advantage of using surfactants instead of crystals for oil foam formation is that oil foam can be produced at lower concentrations of foam stabilizer and at room temperature, without requiring any heating or cooling steps to induce the formation of crystals. However, these oil foams typically remain stable only for a few hours to several days. Long-term stabilization is achieved only at high surfactant concentrations, where crystallization of the surfactants (sorbitan esters, sucrose esters and citric acid ester) upon cooling provides a solid-like network that reinforces the foam structure (Fameau and Binks, 2021).

With respect to a cleaner label product, proteins hold significant potential as foaming agents for the production of oil foams due to several compelling advantages. First, their nutritional value is well-established and widely recognized, aligning with consumer demand for healthier food options. Moreover, in aqueous foams, proteins such as dairy proteins are well known to be very good foam stabilizers: They stabilize gas bubbles by adsorbing at the air–water surface and additionally enhance the stability of the foam liquid channels due to the presence of aggregates (Ho et al., 2022; Murray, 2020). However, proteins cannot be used directly in an oil phase due to their hydrophilic nature. To introduce dairy proteins into oil, de Vries et al. (De et al., 2017) and Feichtinger et al. (2022) used a solvent exchange procedure. To strengthen the interactions between the protein particles and to thus increase the gel strength of the obtained oleogel, a small amount of water can be added to induce capillary forces (de et al., 2018; Feichtinger et al., 2025). The use of aerogel particles based on pea or dairy proteins have also been shown to be an efficient strategy to obtain oleogels from proteins (Plazzotta et al., 2020, 2025; Basso et al., 2026). A recent study also demonstrated that submicron-sized pea protein isolate particles form structured aggregates in sunflower oil, which can generate a space-spanning network at sufficiently high particle concentrations and lead to oleogel formation (Holdstock et al., 2025). While these studies show that hydrophilic protein particles can be used to structure oils by network formation to obtain oil-based gels, it is not known whether protein can also be used as a foaming agent to obtain oil foams.

By incorporating protein particles into the oil, it may be possible to enhance foam stability, while also improving the nutritional profile of the product and aligning with clean-label trends. In oil foams, protein particles could enhance foam stability through multiple mechanisms: 1) by adsorbing on the air-oil surface and decrease the surface tension, and 2) by increasing the viscosity of the oil phase and decrease the oil drainage (Fameau and Salonen, 2014). To the best of our knowledge, there have been no studies exploring the use of dairy proteins to enhance the stability of oil foams.

The aim of this study is to address this knowledge gap by investigating the behavior of various dairy proteins powders in sunflower oil – either alone or combined with a model surfactant (sorbitan monooleate) as additional surfactant – and to establish the relationship between various characteristics of these protein powders with foaming properties. We used six different types of dairy powders to study the effect of the nature of the protein (whey protein *versus* casein micelle), the composition of the powder (presence of lactose and milk fat) and the protein content on the oil foam properties. In addition, we investigated foam formation and stabilization in other vegetable oils (linseed oil and roasted sesame oil), which differ in viscosity, polarity, and fatty acid composition. To explain the differences observed in foam stability over time, we characterized the dairy powders in oil by analyzing particle size, system water content, viscosity, floatability and surface tension, as well the density and the amount of occluded air in the dry powder. For the dairy powder that produced the most stable oil foams at room temperature, we further evaluated foam stability over time across different temperatures.

## 2 Materials and methods

### 2.1 Materials

Sunflower, linseed and roasted sesame oils were purchased from a local supermarket (Cora, France) and used without further purification. The nonionic surfactant sorbitan monooleate, (synthesis grade, purity 65.0 - 88.0 %) was purchased from Sigma-Aldrich, Inc. (Saint Louis, MO, USA). Dairy powders were supplied by Ingredia SA (Arras, France). All the powders were obtained by spray-drying. A detailed description of powder compositions and particle sizes are shown in Table 1. All data were provided by Ingredia.

**Table 1:** Dairy powder composition and dry powder characterization.

Type of proteins	Whey protein isolate	Micellar casein isolate	Milk powder (80/20 CN/WP)	Milk powder (80/20 CN/WP)	Milk protein concentrate (80/20 CN/WP)	Milk protein isolate (80/20 CN/WP)
Abbreviation	WP	CN	CN/WP 26% fat + lac	CN/WP 42% fat + lac	CN/WP lac	CN/WP
Protein (%)	87.75	85.15	26.88	20.33	62.45	83.08
Lactose (%)	3.04	1.48	37.54	31.11	24.9	4.26
Fat (%)	0.03	0.05	26.00	42.50	0.25	0.25
Ash (%)	2.26	7.33	5.80	4.40	7.44	7.32
Moisture (%)	6.19	6.31	3.83	3.03	5.04	4.34
D [4;3] (μm)	45.1	72.5	102.0	166.0	70.7	151.0

### 2.2 Oil foam preparation

10 wt% of sorbitan monooleate was mixed with vegetable oil and left overnight under magnetic stirring at room temperature. Then, the protein powder was added in various concentrations (10 to 30 wt%) directly to the mixture and mixed with an overhead stirrer equipped with a 3.5 cm dissolver impeller (Figure SI1a) during 3 min at 940 rpm at room temperature in a 100 mL beaker. Afterwards, the dispersion was transferred to a glass tube of 31 mm external diameter and 78 mm height, and the dispersion was whipped with a home-made design stirrer during 2 min at 2750 rpm at room temperature (Figure SI1b). To guarantee a homogeneous mixing and whipping, the recipients were moved up and down during the procedures. All the samples were made in triplicate and the oil foams were stored at 5, 20 and 60 °C.

### 2.3 Oil foam characterization

#### 2.3.1 Oil drainage and foam height

Photographs of the tubes containing the oil foam samples were taken with a Poco X3 NFC phone (Xiaomi) at time zero, after 1 h, 24 h, 1 week, 1month and 2 months (when long-term stabilization was obtained). The amount of oil drainage, powder sedimentation and the evolution of foam height with time was determined by the naked eye (phase separation) and also measured by image analysis using ImageJ software (Version 1.54g).

#### 2.3.2 Bubbles characterization

The obtained oil foams were characterized with a ZEISS Axioscope 5 optical microscope, with an Axiocam 208 camera (Zeiss, Germany). We also used a dynamic foam analyzer (DFA100- KRUSS, Germany) to determine the evolution of the mean bubble area with time. The oil foams were produced as described in section 2.2 using sunflower oil with 10 wt% surfactant and 30 wt% dairy powder, and then transferred to the glass column of the DFA100 (internal diameter of 40 mm) in which a prism was incorporated along the entire length of the column. Pictures of the foam were taken every 30 min (up to 24 h) with the camera of the DFA100 instrument (Schneider kreuznach, Germany) with a scanning area of 10.5 × 7.5 mm, positioned at a column height of 55 mm. The software used for images capture was the ADVANCE software, version 1.15-01 (KRUSS, Germany). The resulting high-contrast images were processed (conversion to grayscale with a RenyiEntropy threshold) using ImageJ Software (Version 1.54g) to calculate the evolution of the mean bubble area with time.

### 2.4 Dairy powder characterization

#### 2.4.1 Microstructure observation

Surface morphology and structural topology of dairy powders were investigated, using a JEOL JSM-IT210(LV) high-resolution field-emission scanning electron microscope (SEM) (JEOL Ltd., Tokyo, Japan). The scanning voltage was set to 15 kV. To prepare the samples, the powders were dusted onto a double-sided adhesive carbon tape attached to an inox substrate and sputter-coated with gold using an argon plasma using a Leica EM ACE200 equipment (Danaher, Washington, United States).

#### 2.4.2 Particle size determination in oil

After protein powders were dispersed in sunflower oil containing surfactant as described above, the particle size distributions of dispersions prepared with 30 wt% protein in sunflower oil were determined by static light scattering (Malvern Mastersizer 3000+ with a Hydro SM dispersion unit, Malvern Instruments Ltd, Worcestershire, UK) based on the Mie theory. Sunflower oil was used as the dispersing medium during the measurements, with the stirring speed set to 2000 rpm. Values used for the refractive indices were 1.47 for sunflower oil and 1.54 for protein. Measurements were performed in triplicate, and results are reported based on volume distributions (D[4;3]).

#### 2.4.3 Powder bulk density and occluded air

The bulk density, or apparent density (D_p_), is the density of the powder including the occluded air and was calculated by measuring the true volume (volume enclosed by powder's outer surface, *V*) of a defined mass (*M*) of powder (D_p_ = M/V). The true volume was determined by measuring the pressure change of helium in a calibrated volume using a ULTRAPYC 1200e 6 St2 Quantachrome (Micromeritics Instrument Corporation, USA).

The occluded air content (air within the particle, V_oa_) was calculated according to the GEA Niro method, (Niro). Voa=100Dp−100Ds, where *D*_*s*_ is the theoretical density of powder solids and, for milk, is defined as:

(1) Ds=100%F0.94+%SNF1.52+%W

where *%F* is the fat content, *%SNF* is the solid non-fat content and *%W* is the moisture content.

#### 2.4.4 Powder floatability

0.5 g of dairy powder was carefully placed on the surface of 10 mL of sunflower oil containing 10 wt% surfactant at room temperature in a glass vessel. The floatability of the powder was evaluated by measuring the time required for the particles to sink completely into the oil. This process was recorded by photographing the glass vessels at different time intervals using a Nikon digital camera D850 equipped with an AF-S Micro NIKKOR 60 mm f/2.8G ED lens. We also calculated the dimensionless Bond Number (B_o_) known to quantify the ratio between the gravitational and the surface tension forces acting on the particle and defined as Bo=ΔρgL2γ where Δ*ρ* is the difference in density of the two phases; *g* is the gravitational acceleration; *L* is the particle diameter and *γ* is the surface tension. If B_o_ ≪ 1 the surface tension forces dominate over gravitational forces and the particle tends to remain at the liquid/air interface rather than sinking (Pitois and Rouyer, 2019).

### 2.5 Oil phase characterization

#### 2.5.1 Viscosity of oil-based protein dispersions

The apparent viscosity of sunflower oil with 10 wt% surfactant without and with 10 wt% and 30 wt% of protein particles was measured with an AR 2000ex Rheometer (TA Instruments, Guyancourt, France). The geometry used was a cone/plate (4°, diameter = 6 cm, gap = 117 μm). The experiments were conducted at 20 °C varying the shear rate from 0 to 150 s^−1^ during 1 min. The bulk viscosities were used to explain the foamability and foam stability of the different systems. The measurements were made in triplicate.

#### 2.5.2 Determination of the water content by Karl-Fischer titration method

We used the volumetric Karl-Fischer (KF) titration method to determine the water content in the oil phase, using the C20 Coulometric KF Titrator (Mettler Toledo, Switzerland) and the Karl Fischer titrant (HYDRANAL™ - Composite 5, Honeywell Fluka™, Germany). The sunflower oil containing 10 wt% surfactant, as well as the supernatant obtained after centrifugation (20,000 g for 2 h at 20 °C) of a 10 wt% powder-in-oil dispersion with surfactant, was loaded into a 2 mL disposable syringe (Injekt, Braun, Fisher Scientific, USA) fitted with a metallic needle (0.40 mm diameter, 80 mm length; Sterican, Braun, Fisher Scientific, USA). The weight of the sample inserted in the chamber was measured, and the water content was determined by the instrument in %. The measurements were repeated three times, and the standard deviation was determined.

#### 2.5.3 Surface tension

The air–oil surface tension was measured using an automated surface tension plate reader (Kibron Delta-8, Kibron, Finland) equipped with DyneProbes. For each measurement, 50 μL of dispersion was dispensed onto a 96-well plate. Calibration was carried out by filling the first row of the plate with Milli-Q water at room temperature, following the manufacturer's instructions. All samples were measured in triplicate after a 10 min equilibration period to ensure stabilization of the air–oil surface. To determine the surface tension of the oil phase after powder addition, the supernatant obtained from a 10 wt% powder-in-oil dispersion containing surfactant was used. The dispersion was centrifuged at 20,000 g for 2 h at 20 °C prior to analysis. Measurements were performed in triplicate.

### 2.6 Statistical analysis

All the experiments were carried out in triplicate and the results were expressed as the mean ± standard deviation. The results were compared by one-way analysis of variance and Tukey's test to analyze statistical differences (p < 0.05). The analysis was performed using the Origin2025 program (OriginLab Corporation, Massachusetts, USA).

## 3 Results and discussion

### 3.1 Effect of protein type on oil foam production and stability

First, we attempted to produce oil foams using only protein powders dispersed in sunflower oil. However, irrespective of the protein powder used, the systems consisted mainly of solid particles dispersed in the oil phase, with almost no bubble formation (Figure SI2). This indicates that the proteins did not adsorb at the air–oil surface and were therefore unable to stabilize air bubbles. In addition, measurements of the air–oil surface tension for pure sunflower oil and for oil containing the different dairy powders showed no significant differences, further confirming that no surface-active species were present under these conditions (Table SI1). As the protein powder alone was not able to stabilize air bubbles, we employed 10 wt% of nonionic surfactant sorbitan monooleate, as a model lipophilic surfactant to stabilize the air bubbles and facilitate oil foam formation, and subsequently investigated the effect of adding protein powder on the resulting surfactant-stabilized foams (Liu and Binks, 2022). The mechanism by which this surfactant generates oil foams remains unknown. Liu and Binks proposed that a surface-active complex form through hydrogen bonding between the carbonyl groups of the triglycerides and the hydroxyl groups of the sorbitan monooleate (Liu and Binks, 2022). Moreover, this relatively high concentration was chosen to ensure high foamability and adequate foam stability over several hours, allowing us to better investigate the effect of adding dairy powder to the system.

Without protein particles, oil drainage occurred quickly after foam formation: 60 % of oil was drained after 1 h, and the foam completely disappeared after five days (Figure SI3). This result is in accordance with previous results obtained on oil foam stabilized by sucrose and sorbitan ester surfactants (Liu and Binks, 2021b, 2022). To increase the stability, we started by investigate the impact of different types of dairy protein on foam stabilization: (1) whey protein (WP), (2) casein micelles (CN), (3) a powder containing a mixture of casein micelles and whey proteins at a ratio of 80/20, respectively, with 26 % of milk fat and 37.5 % of lactose (CN/WP 26 % fat + lac) and (4) a powder containing a mixture of casein micelles and whey proteins at a ratio of 80/20, respectively, with 42 % of milk fat and 31.1 % of lactose (CN/WP 42 % fat + lac) (Table 1). To further investigate the effect of different powders, we examined the influence of powder composition: (5) a powder composed of a mixture of casein micelles and whey proteins at an 80/20 ratio, respectively, containing 24.9 % lactose (CN/WP lac), and (6) a powder composed of a mixture of casein micelles and whey proteins at an 80/20 ratio, respectively (CN/WP). All the oil foams were studied at powder concentrations ranging from 0 to 30 wt%. As the powders had different compositions, the amount of protein present in the oil foams varied between powders (Table SI2).

**Fig. 1:**
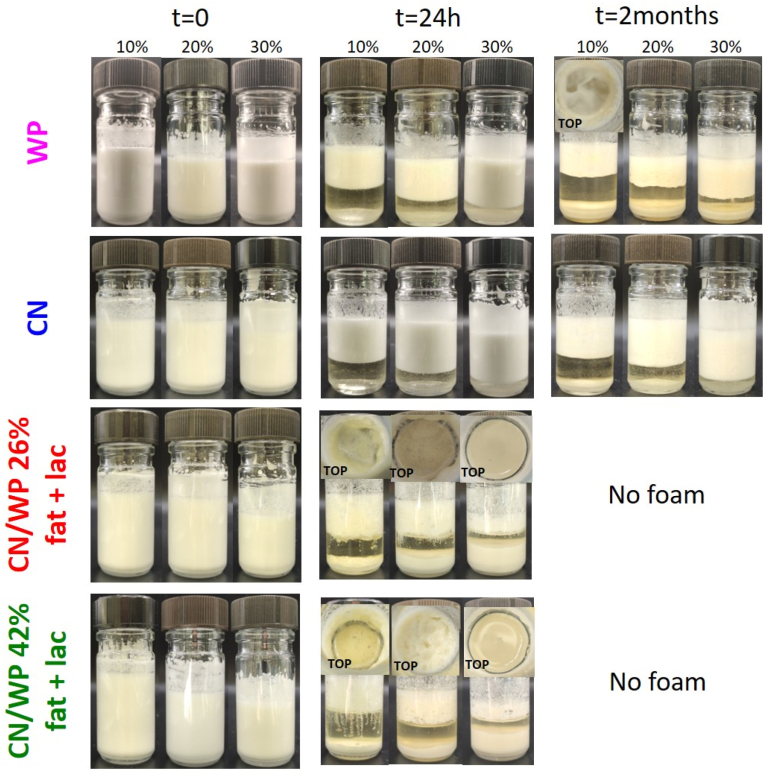
Photographs of sunflower oil foams based on 10 wt% surfactant with the addition of varying concentrations of protein powders from 10 to 30 wt% over time: WP (first row), CN (second row), CN/WP 26% fat + lac (third row) and CN/WP 42% fat + lac (fourth row). Additional photos were taken from the top of the foam to show that the foams had completely collapsed in those samples (TOP). When the foam disappeared completely due to destabilization, we labeled the figure as “No Foam.”

**Fig. 2:**
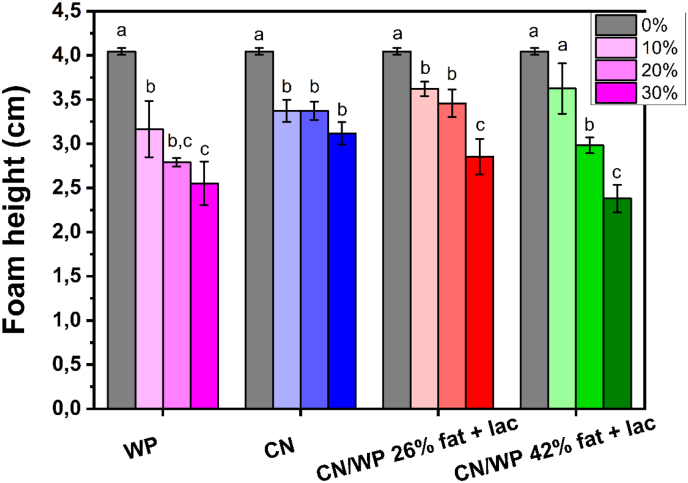
Foam height measured just at the end of the foaming process in centimeters for the different dairy powders at different concentration from 0 (only surfactant) to 30 wt% of dairy powders: whey proteins (WP) in pink, casein (CN) in blue, 80 % casein and 20 % whey protein with 26 % fat and 37 % lactose (CN/WP 26% fat + lac) in red, and 80 % casein and 20 % whey protein with 42 % fat and 31 % lactose (CN/WP 42% fat + lac) in green. The small letters a–c indicates groups of statistical differences according to Tukey's test (p < 0.05) for each powder as a function of the concentration from 0 to 30 wt%.

For the influence of the type of protein, we measured the obtained foam height immediately after foaming. Foams were obtained for all samples, independent of both the protein powder composition and its concentration (Fig. 1, Fig. 2). In the presence of 10 wt% protein powder, the foam height was approximately 3.0–3.5 cm whatever the protein powder used, which was lower than that of the oil foam stabilized solely by the surfactant (foam height = 4 cm, Fig. 2). For higher concentrations of protein powder, the initial foam height decreased further. This reduction in initial foam height can be attributed to an increase in the viscosity of the bulk medium with increasing protein powder concentrations in comparison to the oil and surfactant system, which hampered the processes of air entrapment and bubble breakage (Figure SI4) (Politova et al., 2018). Thus, despite the high protein powder content and the associated increase in viscosity, foam production remained possible. Therefore, the added powders did not hinder foam formation or the surfactant-driven stabilization of small bubbles.

**Fig. 3:**
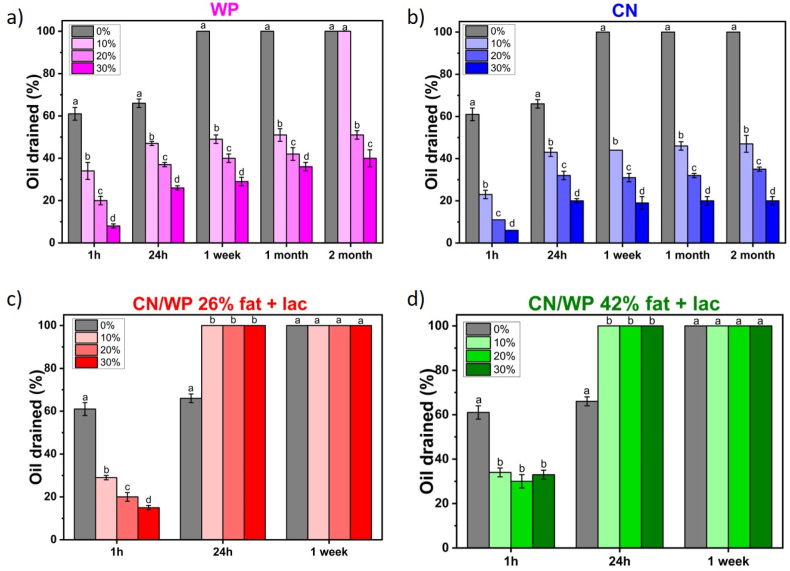
Percentage of oil drained over time at room temperature for sunflower oil foams based on 10 wt% surfactant with the addition of protein powders at different concentrations from 0 to 30 wt% as a function of time: (a) WP, (b) CN, (c) CN/WP 26% fat + lac and (d) CN/WP 42% fat + lac. When the foam was completely destabilized it corresponds to 100 % of oil drained. The small letters a–d indicates groups of statistical differences according to Tukey's test (p < 0.05) for each time as a function of the concentration from 0 to 30 wt%.

After foam production, the oil foams were stored at room temperature. We followed the foam stability by measuring the evolution of the oil drainage over time (Fig. 1, Fig. 3). Compared to the foam stabilized solely by the surfactant (60 % oil drained after 1 h), we observed a significant reduction in drainage within the first hour after foam formation when at least 10 wt% of protein powder was added: around 30 % of oil drained for WP, CN/WP 26% fat + lac and CN/WP 42% fat + lac and around 20 % for CN. To understand the differences in the oil drainage, we compared the bulk viscosity between the pure oil-surfactant systems and when we added the protein powders since a high bulk viscosity is known to enhance foam stability by slowing down liquid drainage (Drenckhan and Saint-Jalmes, 2015) (Figure SI4). For all the systems, the addition of protein powder led to an increase of bulk viscosity, which increased the foam stability against oil drainage with increasing powder concentration. Only in the case of CN/WP 42% fat + lac, an increase of the powder concentration did not further decrease the oil drainage, even if the bulk viscosity increased in comparison to the pure oil-surfactant system. The foam stability is not only linked in the reduction of oil drainage.

For both whey protein (WP) and casein (CN), oil drainage was most pronounced during the first hour after foam production, then progressively slowed over time, reaching 40 % and 20 % oil drained after 2 months at 30 wt% powder, respectively (Fig. 3a and b). For these samples, a substantial amount of foam remained after two months of storage (Fig. 1). Notably, no powder sedimentation was observed after 24 h for either WP- or CN-stabilized oil foams. However, after two months of storage at room temperature, WP foams at 10 wt% underwent complete collapse. At higher concentrations (20 and 30 wt%), WP foams showed significant sedimentation, although foam was still present. In contrast, CN-based foams remained stable at all concentrations after two months, displaying only a very thin sediment layer at the bottom (Fig. 1).

For the other powders containing milk fat and lactose (CN/WP 26% fat + lac and CN/WP 42% fat + lac), all foams collapsed within 24 h, regardless of powder concentration (Fig. 1, Fig. 3c–d). Even though these powders also initially slowed down oil drainage, the presence of high levels of fat and lactose actually decreased foam stability over longer time: After 24 h, only 60 % oil had drained for foams with surfactant only, while full drainage and foam collapse were reached for the powders containing fat and lactose. Overall, among the four dairy powders studied, CN produced the most stable foams over time (Fig. 1, Fig. 3).

**Fig. 4:**
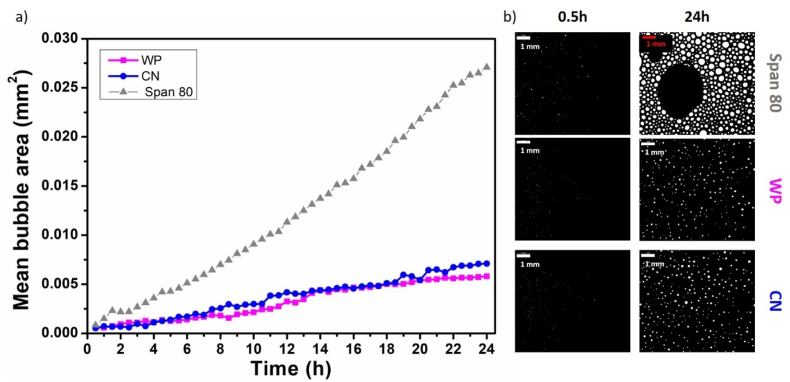
(a) Evolution of the mean bubble area over time (hours) at room temperature for oil foams with only surfactant at 10 wt% (grey triangle) and with addition of 30 wt% of WP (pink square) or CN (blue circle); b) respective images of the foams at different times: 0.5 h and 24 h.

For the most stable foams formulated with casein (CN) and whey protein (WP) at 30 wt%, we followed the evolution of air bubbles over 24 h at room temperature by measuring the mean bubble area through image analysis (Fig. 4). It is important to note that immediately after foam production, the bubbles could not be detected by the imaging system with a lower detection limit of 50 μm in diameter. This indicates that all oil foams, whether or not they contained dairy proteins, initially consisted of very fine bubbles smaller than 50 μm. Significant differences emerged over time. In the case of oil foams stabilized solely by the surfactant, rapid bubble growth was observed, with the mean bubble area increasing sharply within the first hours. This behavior is consistent with known accelerated coarsening and coalescence processes in the presence of small molecular weight surfactants in the absence of additional stabilizing agents (Drenckhan and Saint-Jalmes, 2015).

In contrast, the oil foams containing WP or CN powder exhibited a much slower increase in mean bubble area throughout the 24 h-period. After 24 h, the mean bubble area in both dairy powder systems was approximately 0.005 mm^2^, which is approximately five times smaller than the bubble area observed in the surfactant-stabilized foams (approximately 0.027 mm^2^, Fig. 4a). Both dairy protein-stabilized foams showed similar trends, effectively preserving smaller bubble sizes over time. These results clearly demonstrate the dual stabilizing role of CN and WP in oil foams. First, they contribute in reducing oil drainage due to the high viscosity in the presence of the powder; second, by being present between the air bubbles in the bulk phase, they reduce bubble contact and coalescence. The similar performance of WP and CN suggests that both protein types provide comparable structural reinforcement to the foam in the first 24 h.

Since the protein content varies between the different powders, additional oil foams with standardized protein content were prepared, to investigate whether foam stabilization was primarily due to the amount of proteins themselves, or due to the total dry matter content (Table 1 and Table SI2). This comparison was crucial, as our previous observations showed that foams made with powders containing lower protein content and higher amounts of milk fat and lactose were unstable (CN/WP 26% fat + lac and CN/WP 42% fat + lac). As shown in Table SI2, for 30 wt% dairy powder (most stable foams), the protein concentration was around 26 % for WP and CN, but only 6.10 % for CN/WP 42% fat + lac. To evaluate if the good performance of WP and CN is due to their high amount of protein, the powder concentration was adjusted to the lowest level so that all samples contained 6.10 wt% of protein (the one tested previously for CN/WP 42% fat + lac), i.e. 6.95 wt% and 7.16 wt% powder of WP and CN, respectively.

As shown in Figure SI5, foams containing whey protein (WP) destabilized after one week, whereas foams produced with casein (CN) were still present one month after foam production. Therefore, the lack of foam stability is most likely not due to a lower protein content, but rather to differences in overall powder composition (presence of milk fat and/or lactose). Overall, these results highlight that dairy powder composition plays a key role in ensuring long-term foam stability.

### 3.2 Effect of lactose and milk fat on foam stability

**Fig. 5:**
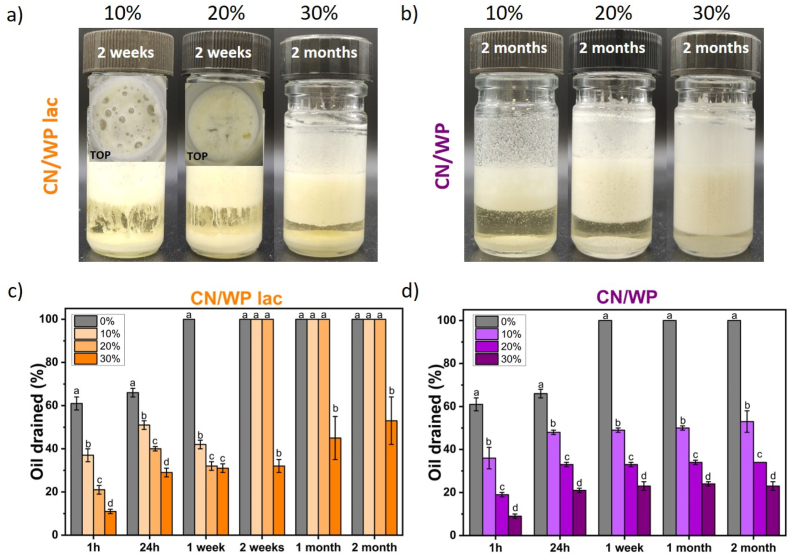
Appearance of oil foams over time at varying powder concentrations for: (a) CN/WP lac and (b) CN/WP. Additional photos were taken from the top of the foam to show that the foams had completely collapsed in those samples (TOP). For all the concentrations, the foams produced with CN/WP were more stable than the ones obtained with CN/WP lac. (c-d) Percentage of oil drained with time at room temperature for sunflower oil foams based on 10 wt% surfactant with the addition of protein particles for the different dairy powders at different concentration from 0 to 30 wt% of dairy powders: (c) CN/WP lac and (d) CN/WP. The small letters a–d indicates groups of statistical differences according to Tukey's test (p < 0.05) for each time as a function of the concentration from 0 to 30 wt%.

Since the unstable oil foams were produced with dairy powders containing both lactose and milk fat, we aimed to identify which component was primarily responsible for the observed foam instability: lactose or milk fat. We studied two additional dairy powders with the same CN/WP ratio of 80/20: one powder containing 24.9 % lactose, but almost no milk fat (0.25 %, CN/WP lac), and another with a very small amount of lactose (4.3 %) and almost no milk fat (0.25 %, CN/WP), as shown in Table 1 (Fig. 5a and b). Similar to the previously tested powders, the initial foam height with 10 wt% of either powder was slightly lower (∼3 cm) compared to that of the foam formed with surfactant alone (∼4 cm, Figure SI6), but remained unchanged at higher powder concentrations. The decrease of foam height was due to an increase of viscosity in the presence of the powders (Figure SI4). This higher bulk viscosity decreased the amount of entrapped air as explained before.

In the absence of fat (CN/WP lac), foams prepared with 10 wt% and 20 wt% powder showed destabilization after two weeks (Fig. 5a–c). In contrast, at the higher powder concentration of 30 wt%, the foams remained stable for at least two months. However, powder sedimentation started after around one month (Fig. 5a). These results show that the absence of fat increases the stability of the foams, i.e. fat destabilizes the foam. When also the lactose content was limited (CN/WP), the foam remained stable for at least two months without powder sedimentation, independent of the concentration used (Fig. 5b–d). Oil drainage decreased at higher protein concentrations as already observed for the powders discussed in section 3.1 (Fig. 5c–d). Without fat and lactose, the CN/WP powder stabilized the foam as effectively as observed for CN and WP powders one (Fig. 1). These results indicate that lactose, although less detrimental than milk fat, also contributes to foam destabilization, particularly at lower powder concentrations. Removal of fat and lactose from the dairy powder thus increased foam stability.

**Fig. 6:**
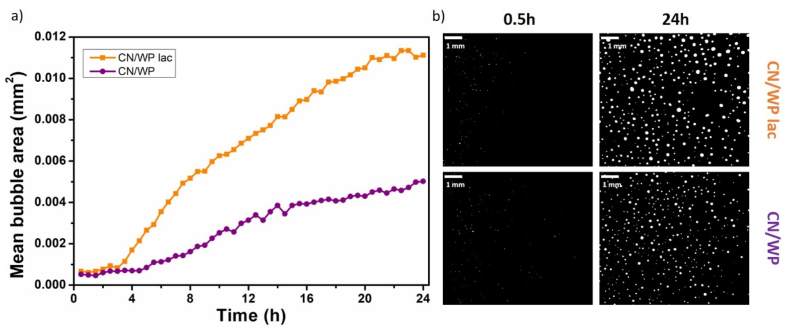
(a) Evolution of the mean bubble area at room temperature over time for oil foams produced with 30 wt% of CN/WP lac in orange squares and CN/WP in purple circles; and (c) corresponding pictures of the foams at different time after foam production: 0.5 h and 24 h.

This trend in oil foam stability was further supported by monitoring the evolution of the mean bubble area over time. After 24 h, the mean bubble area in foams made with 30 wt% CN/WP and lactose was approximately 0.011 mm^2^, roughly twice than that of foams made with CN/WP (∼0.005 mm^2^, Fig. 6), indicating more rapid bubble coalescence and less effective stabilization for lactose-rich powder. Without fat and lactose, the bubble was similar to that of CN and WP powders separately (∼0.005 mm^2^ after 24 h, Fig. 4a). These observations confirm that powder composition plays a critical role in determining foam stability further highlighting the key role of fat and lactose on oil foam stability in terms of bubbles size evolution.

### 3.3 Effect of the nature of the vegetable oils

To validate the observations made with sunflower oil, we extended the study to two additional edible oils: sesame and linseed oils. These oils differ in composition (main fatty acid) and physical properties as the interfacial tension (Cong et al., 2020), enabling evaluation of whether the previously observed trends depend on the oil type. These oils were selected to cover a wide range of unsaturation: sunflower oil (high ω-6), linseed oil (high ω-3), and roasted sesame oil (higher monounsaturated fraction and richer in minor surface-active compounds) (Ji et al., 2019). Foams prepared with surfactant alone reached higher foam heights with sesame and linseed oils (≈5 cm) compared to that with sunflower oil (≈4 cm, Figure SI7a). For both sesame and linseed oil, a large amount of foam was still present after one week, whereas no more foam was observed for sunflower oil-based foam (Figure SI7c). These results align with earlier work of Liu and Binks, who demonstrated that the oil type affects the foamability and foam stability in the case of surfactants, though without offering an explanation (Liu and Binks, 2021a). However, the differences observed among the three oil foams are likely due to variations in oil composition, viscosity, surface tension, and minor polar components (e.g., free fatty acids, mono-/di-glycerides, phospholipids) (Liu and Binks, 2022). Future studies on surfactant-based oil foams should further investigate the influence of these oil physicochemical properties. Such effects have already been extensively studied at oil–water interfaces and shown to play a key role in emulsion stabilization, and similar mechanisms are expected to be important for the formation and stability of oil foams (Rahman and Eastoe, 2022; Hosseinpour et al., 2021).

**Fig. 7:**
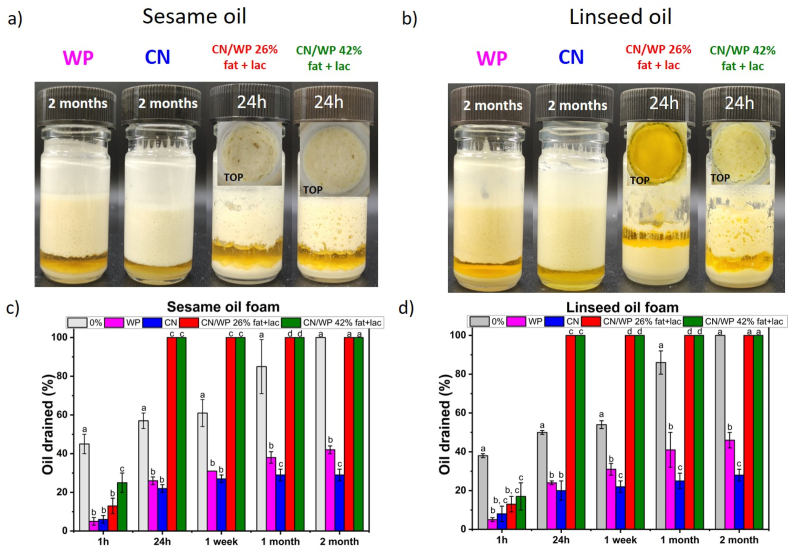
Appearance of the oil foams for 30 wt% WP and CN after two months of storage and after 24 h for 30 wt% CN/WP 26% fat + lac and CN/WP 42% fat + lac in the different oils: (a) sesame oil and (b) linseed oil. The corresponding oil drained in % with time for the different oil foams are presented for: (c) sesame oil and (d) linseed oil. The small letters a–d indicates groups of statistical differences according to Tukey's test (p < 0.05) for each time as a function of 30 wt% dairy powder nature.

When dairy protein powders (30 wt%) were added, foam heights just after foam production for all the oils converged to around 3–3.5 cm (Figure SI8). As observed previously, powders containing lactose and fat yielded unstable foams, which collapsed within 24 h regardless of the oil used (Fig. 7a and b). In contrast, foams stabilized with WP and CN powders exhibited excellent stability in both sesame and linseed oils, maintaining their structure and appearance for at least 2 months at room temperature (Fig. 7a and b). After this period, oil drainage remained limited (approximately 45% and 30% for WP- and CN-stabilized foams, respectively, in both oils; Fig. 7c–d), consistent with the results observed in sunflower oil (Fig. 3). As previously noted for sunflower oil, WP-stabilized foams showed sedimentation after two months, whereas no sedimentation was observed for CN-stabilized foams. These results confirm a consistent trend across all tested oils: powders containing fat and lactose destabilize foams over time, while WP and CN provide long-term stability, with CN consistently outperforming WP. In contrast to the oil foams stabilized just with the surfactant, the performance of the protein powders was largely independent of the oil type, consistently yielding similar foam height after foam production (foamability) and stability outcomes across sunflower, sesame, and linseed oils probably due the presence of protein particles that overcomes the oil type properties. These three oils provide a relevant comparison to assess the robustness of the observed powder behavior across oils of different composition and interfacial characteristics.

### 3.4 Link between oil foams stability and protein particles properties

To gain deeper insight into the differences observed between the oil foams obtained with the six protein powders in sunflower oil, we investigated and compared several key parameters which could influence dairy powder functionality during foam production. The considered properties of the protein powder dispersions were: residual moisture content, surface tension, particle size, agglomerate morphology and floatability, Table 2. Also, the powder apparent density and the volume of occluded air present in the powder were measured and the Bond Number was calculated (Table 2).

**Table 2:** Physical characterization of the system containing protein powder, 10 wt% sorbitan monooleate and sunflower oil.

	Stable foam			Unstable foam		
	WP	CN	CN/WP	CN/WP 26% fat + lac	CN/WP 42% fat + lac	CN/WP lac
**Water content in oil phase (%)**	0.153 ±0.001^a^	0.108 ±0.001^b,d^	0.088 ±0.005^c^	0.100 ±0.001^d^	0.102 ±0.004^b,d^	0.108 ±0.002^b^
**Surface tension (mN/m)**	35.3 ± 0.9^a^	34.4 ± 0.4^a^	34.8 ± 0.6^a^	35.0 ± 1.0^a^	35.1 ± 0.5^a^	34.2 ± 0.4^a^
**D[4;3] (μm) in oil**	65.8 ± 0.7^a^	90.7 ± 0.9^b^	119±3^c^	121±2^c^	153±3^d^	87.0 ± 1.0^b^
**Powder apparent density (g/mL)**	0.407	0.363	0.344	0.453	0.449	0.483
**Volume powder occluded air (mL/100 g)**	36.0	44.8	40.5	7.3	4.7	18.9
**Bond Number**	0.030	0.083	0.373	0.138	0.370	0.062

^a-d^ The small letters indicates groups of statistical differences according to Tukey's test (p < 0.05) for each powder.

As water was present in all the powder and could eventually be released to the oil phase and modify the surfactant behavior, we quantified the amount of water present in bulk systems containing 10 wt% surfactant and 10 wt% dairy powder. The results showed that powders producing unstable foams such as CN/WP 26% fat + lac, CN/WP 42% fat + lac, and CN/WP lac, contained about 0.1 % water in the oil phase (Table 2). Interestingly, CN powders also exhibited a similar water content yet still produced stable foams. Another parameter that moisture could change in the system is the oil surface tension. As showed in Table 2, the different types of dairy protein did not affect the sunflower oil surface tension and remained similar to the pure oil (34.1 mN/m). The water content in the bulk oil systems was not a parameter explaining the different oil foam stabilization behavior between the powder.

**Fig. 8:**
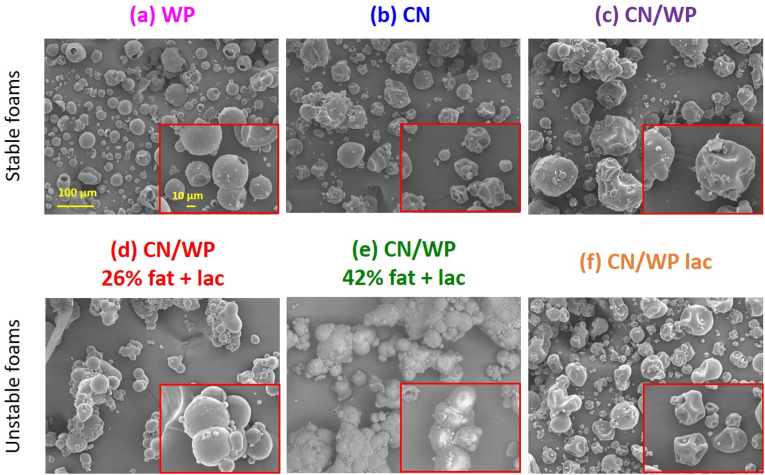
Microstructure characterization of the powders by scanning electron microscopy. Images of dried protein powder are presented for: (a) WP, (b) CN, (c) CN/WP, (d) CN/WP 26% fat + lac, (d) CN/WP 42% fat + lac and (f) CN/WP lac. The insets represent magnified images. The scale bar is the same for all images.

Another possible factor influencing the foam stabilization of the powders is the morphology of the agglomerates once dispersed in oil containing 10 wt% surfactant. Scanning electron microscopy (SEM) was used to visualize the morphology of the dry powders and access potential particle clustering (Fig. 8). WP exhibited typical spherical shape (Fig. 8a) (Sadek, 2015; Palzer et al., 2012). In contrast, CN, CN/WP, and CN/WP lac powders (Fig. 8b, c, and f, respectively) displayed the morphology with irregular surfaces and cavities (Sadek, 2015; Palzer et al., 2012). The most pronounced differences were observed for the milk-fat-containing powders (CN/WP 26% fat + lac and CN/WP 42% fat + lac; Fig. 8d and e, respectively), which formed large agglomerates (Sadek, 2015; Palzer et al., 2012). However, these morphological differences do not explain the observed oil foam instability. Indeed, CN/WP lac has a markedly different morphology from CN/WP 26% fat + lac and CN/WP 42% fat + lac without agglomerate, yet it also produces unstable oil foams. Conversely, CN/WP lac exhibits a morphology closed to that of CN and CN/WP powders, although the latter two powders produce stable oil foams.

We also determined the particles size after dispersion in the sunflower oil containing 10 wt% surfactant (Table 2). WP had the smallest particles (D[4; 3] = 65.8 μm). CN and CN/WP lac showed similar sizes (90.7 and 87 μm), yet only CN formed stable foams. Likewise, CN/WP and CN/WP 26% fat + lac had comparable particle sizes (119 and 121 μm), but only CN/WP produced stable foams. The SEM observations of dry powders were consistent with the particle size data from light scattering showing no changes of size after dispersion in the oil. Overall, neither SEM morphology nor particle size (D[4; 3]) showed a clear correlation with foam stability.

Next to particle size, also the apparent density of the protein powder was determined. As shown in Table 2, the powders that produced stable oil foams (WP, CN, and CN/WP) exhibited lower apparent densities, ranging from 0.344 to 0.407 g/mL. In contrast, powders that produced unstable foams (CN/WP 26% fat + lac, CN/WP 42% fat + lac, and CN/WP lac) had higher apparent densities (between 0.449 and 0.483 g/mL). Because apparent density includes the pore volume of the powder, we also quantified the amount of occluded air. The foams with higher stability showed higher amount of air inside the powder particle. It's worth to say that the occluded air is related to the powder composition and the spray drying technique used to produce the powder (Huppertz and Gazi, 2016; Aguilar and Ziegler, 1994; Hogan and O'Callaghan, 2010; Wang et al., 2021; Zhou et al., 2025).

**Fig. 9:**
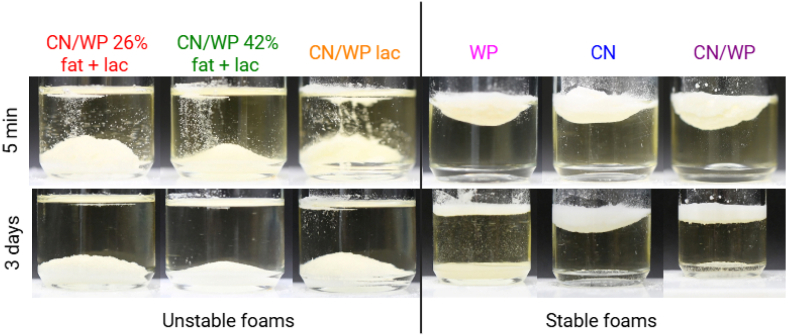
Pictures at 5 min (first row) and 3 days (second row) after the addition of 0.5 g of the different powders at the air/liquid (sunflower oil containing 10 wt% of surfactant) surface to evaluate their wetting behavior. All the powders leading to unstable foams sink rapidly in the liquid (CN/WP 26% fat + lac, CN/WP 42% fat + lac, and CN/WP lac). All the powders leading to stable foams remained at the air/oil surface after 5 min (WP, CN and CN/WP). However, WP sink in the liquid phase after 3 days, whereas CN and CN/WP remained at the air/oil surface.

These results suggest that the density and occluded air of the powders are important and may influence different dynamic aspects: the dispersibility of the particles in oil, the contact between particles and oil, and the sedimentation of the particles. To gain further insight into the wetting and sedimentation behavior of the powders following oil foam formation, the floatability and sedimentation kinetics of each powder were assessed by gently placing the powders at the air/oil surface and monitoring their movement over time. As shown in Fig. 9, for all powders that produced unstable foams (CN/WP 26% fat + lac, CN/WP 42% fat + lac, and CN/WP lac), the protein powder sedimented in the oil phase (oil + surfactant) in few minutes, whereas powders creating stable foams remained on the oil surface during days. Once the particles were dispersed into the oil phase, these powders were unable to remain suspended within the oil bulk to reduce drainage and prevent further bubble coalescence. Instead, they gradually settled at the bottom, as observed in the oil foam images over time (Fig. 1, Fig. 5). This fast sedimentation could come from the presence of particles agglomerates in the case of powders containing fat and lactose as shown by SEM. In contrast, when the powders remained at the air–oil surface, the particles appeared to be better integrated within the foam structure, and no sedimentation was observed. Repeating the experiments in oil without surfactant led to identical results, suggesting that the surfactant has a negligible effect on powder wetting and sedimentation (Figure SI9). This finding demonstrates a direct relationship between foam stabilization and the limited dispersion and sedimentation of particles. It is also noteworthy that the WP powder eventually sedimented within 3 days. This delayed sedimentation likely explains the initial foam stability, followed by a gradual loss of stability over time due to particle settling.

It is well established in the literature that the tendency of particles to float or sediment can be predicted using the Bond Number (B_o_). The B_o_ for all the dairy powders in the oil–surfactant system was calculated and found to be less than one in all cases (Table 2), indicating that the particles should theoretically remain floating. Nevertheless, powders containing fat and/or lactose sank, despite their Bond Numbers being below one. This apparent contradiction could be explained by the fact that these powders likely move collectively under the influence of lateral capillary forces, forming larger aggregates that behave as heavier particles (Vella, 2015). Another plausible explanation is related to powder composition: during spray drying, fat can migrate to the particle surface (Fitzpatrick, 2007; Fitzpatrick et al., 2004), resulting in surface enrichment that alters the interfacial properties and increases the particles’ wettability by oil, thereby promoting sedimentation.

These findings highlight the crucial role of powder composition in determining oil foam stability. Powders rich in milk fat and lactose exhibited higher apparent density and low amount of occluded air, which promoted rapid sedimentation. As a result, they were unable to remain in the bulk oil phase to reduce drainage, leading to poor foam stabilization. In contrast, powders with lower density and higher amounts of occluded air, such as CN and WP, resisted sedimentation and became effectively trapped within the oil phase during foam formation. This entrapment reduced drainage and bubbles coalescence which enhanced long-term foam stability.

## 4 Conclusion

This study presents, for the first time, a simple and effective strategy to produce surfactant-based oil foams with increased stability by using dairy protein particles as additional bulk stabilizers. Although foams could be generated with sorbitan monooleate as a surfactant only, no long-term stability was obtained. We demonstrate that the stability of the oil foams dramatically increased by the inclusion of dairy powders, particularly casein micelle and whey proteins. Therefore, the stabilizing mechanism differs from classical Pickering foams and is instead linked to the ability of dispersed particles to reinforce the foam structure in the continuous oil phase. In these systems, dairy protein particles entrapped air bubbles in the continuous oil phase preventing them against coalescence and reducing oil drainage. Although foam stability is improved by the addition of dairy powders, oil drainage still occurs in all systems, indicating that the formulations require further optimization to fully prevent oil drainage. One way to enhance foam stability in the future could be to combine these particulate-stabilized foams with polymeric structuring agents capable of inducing oil gelation (e.g., ethylcellulose) (Gravelle et al., 2018). It is also important to note that the amount of sorbitan monooleate used in this model system is too high for real food applications. Future work will therefore focus on reducing the surfactant concentration and replacing it with alternative food-grade emulsifiers more suitable for food formulations, such as lecithin or CITREM (Argyri et al).

Foam stability was found to be primarily linked to the sedimentation tendency of the protein powders: rapid sedimentation of dairy particles in the oil phase led to oil-foam destabilization. Differences in sedimentation behavior were strongly related to powder composition, which affected both powder density and the amount of occluded air. In particular, powders containing high levels of milk fat and lactose exhibited higher bulk density and lower occluded air content, which promoted sedimentation and resulted in rapid destabilization and unstable foams. Moreover, milk fat-rich powders showed pronounced particle aggregation, in contrast to powders with low or no milk fat content. The occurrence of these aggregates is also depended on powder composition and was detrimental to foam stability. In contrast, milk fat- and lactose-free powders containing casein and/or whey proteins produced highly stable foams. Importantly, these stability trends were consistent across different vegetable oils (sunflower, sesame, and linseed oils), demonstrating the robustness of the results. To our knowledge, no studies have yet investigated the use of non-interfacially active particles to enhance the stability of surfactant-based oil foams, making this a novel research direction. Based on our findings, future work using controlled model particle systems (with tailored size, shape, density, and porosity) would be particularly valuable to decouple the effects of protein composition from particle physical properties and to further clarify the mechanisms governing stability in these complex systems.

Preliminary temperature stability tests revealed excellent long-term performance at refrigerated and ambient conditions, where foams retained their structure for at least two months (SI.Note 1, Figures SI10-12). However, at elevated temperatures (60 °C), both casein- and whey-stabilized foams rapidly collapsed, indicating that protein particles and surfactants alone cannot prevent destabilization under thermal stress (SI.Note 1, Figures SI10-12). This points to the need for complementary stabilization strategies to lead to high temperature resistance of the oil foams.

Overall, this work establishes that dairy protein powders are versatile, food-grade particulate stabilizers for oil foams, enabling the design of aerated, milk fat-reduced, and nutritionally enhanced products. The method is clean-label, scalable, and adaptable to multiple edible oils, providing a promising alternative to traditional solid fat systems. Protein-stabilized oil foams can contribute to healthier formulations that meet consumer demand for both functionality and nutrition.

## Contributions

Anne-Laure Fameau conceived and supervised the project, contributed to discussing and analyzing the experimental results, and wrote the first draft of the paper; Luisa Azevedo Scudeller carried out the experiments, analyzed and discussed the data, and wrote the first draft of the paper; Annika Feichtinger carried out the experiments and contributed to the writing of the manuscript, Severine Bellayer, and Thierry Six, carried out the experiments; Manon Hiolle, Guillaume Delaplace and Elke Sholten contributed to discussing the experimental results. All authors analyzed and discussed the data and contributed to the writing of the manuscript.

## Declaration of competing interest

The authors declare the following financial interests/personal relationships which may be considered as potential competing interests:FAMEAU Anne-Laure reports financial support was provided by Ingredia Europe. FAMEAU Anne-Laure reports a relationship with Ingredia Europe that includes: funding grants. Dr. Anne–Laure Fameau is an editor of this journal If there are other authors, they declare that they have no known competing financial interests or personal relationships that could have appeared to influence the work reported in this paper.

## Data Availability

The data that support the findings of this study are available from the corresponding author upon reasonable request.
