# Supplementary material for: Oil foam based on dairy proteins particles and surfactant
Source: Curr Res Food Sci. 2026 Feb 7;12:101343. doi: 10.1016/j.crfs.2026.101343 (PMC12917518; doi:10.1016/j.crfs.2026.101343)
Supplement: Multimedia component 1 [file mmc1.docx]

**Supporting Information**

**Oil foam based on dairy proteins and surfactant**

*Luisa Azevedo Scudeller^1^, Annika Feichtinger^2^, Séverine Bellayer^1^, Thierry Six^1^, Manon Hiolle^3^, Guillaume Delaplace^1^, Elke Sholten^2^ and Anne-Laure Fameau^1*^*

^1^ INRAE, University Lille, CNRS, INRAE, Centrale Lille, UMET, 59000 Lille, France.

^2^ Physics and Physical Chemistry of Foods, Wageningen University and Research, Bornse Weilanden 9, Wageningen, 6708 WG, The Netherlands.

^3^ Ingredia – Ingredia Dairy Experts, 62033 Arras, France

Corresponding author's Email: [anne-laure.fameau@inrae.fr](mailto:anne-laure.fameau@inrae.fr)

**Figure SI.1**: (a) Photograph of the mixing device used to disperse the dairy powder in the oil phase and (b) Photograph of the home-made milk foamer setup.


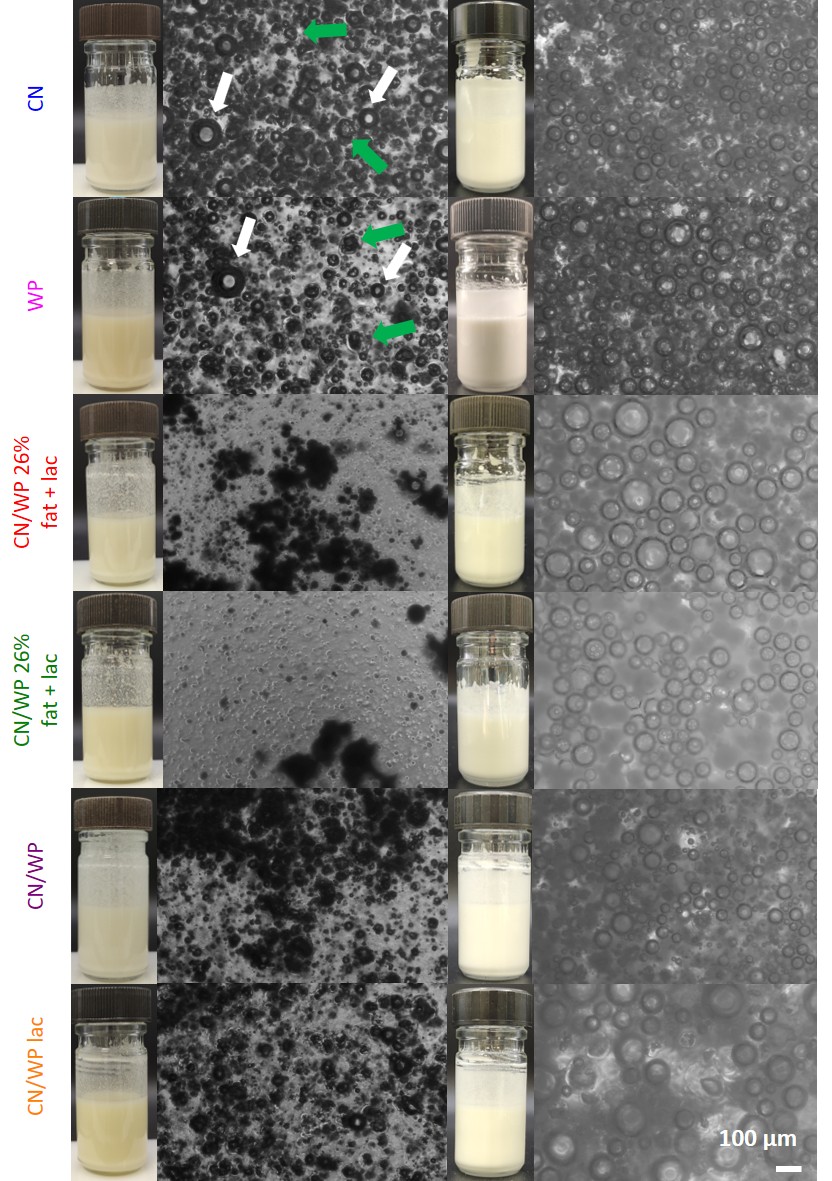


**Figure SI.2**: Photographs of the foams and their corresponding microscopy images taken immediately after foaming the powders in oil without surfactant (first column) and with surfactant (second column). White arrows indicate air bubbles, while green arrows highlight particle agglomerates surrounded by oil. The scale bar is identical across all microscopy images.


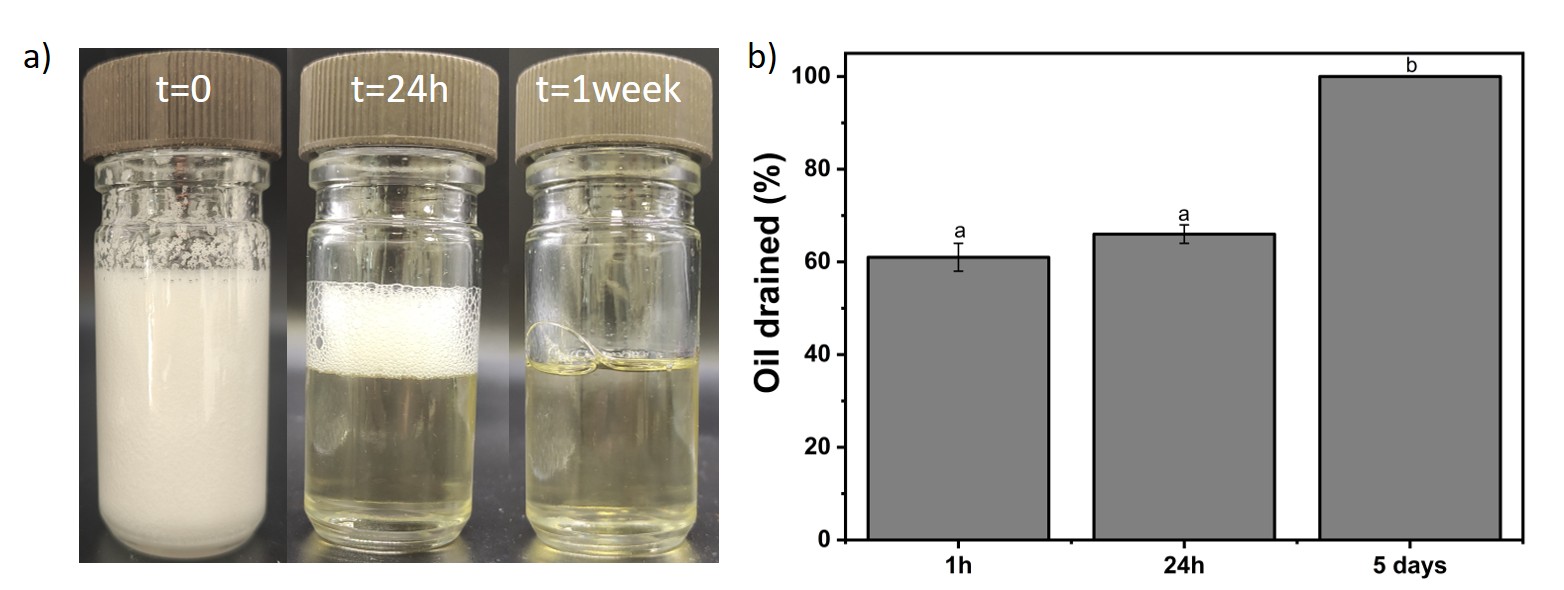


**Figure SI.3**: (a) Photographs of sunflower oil foams based on 10 wt.% surfactant without the addition of protein particles with time at room temperature. (b) Evolution of the oil drained (%) with time. The small letters a–b indicates groups of statistical differences according to Tukey’s test (p < 0.05).


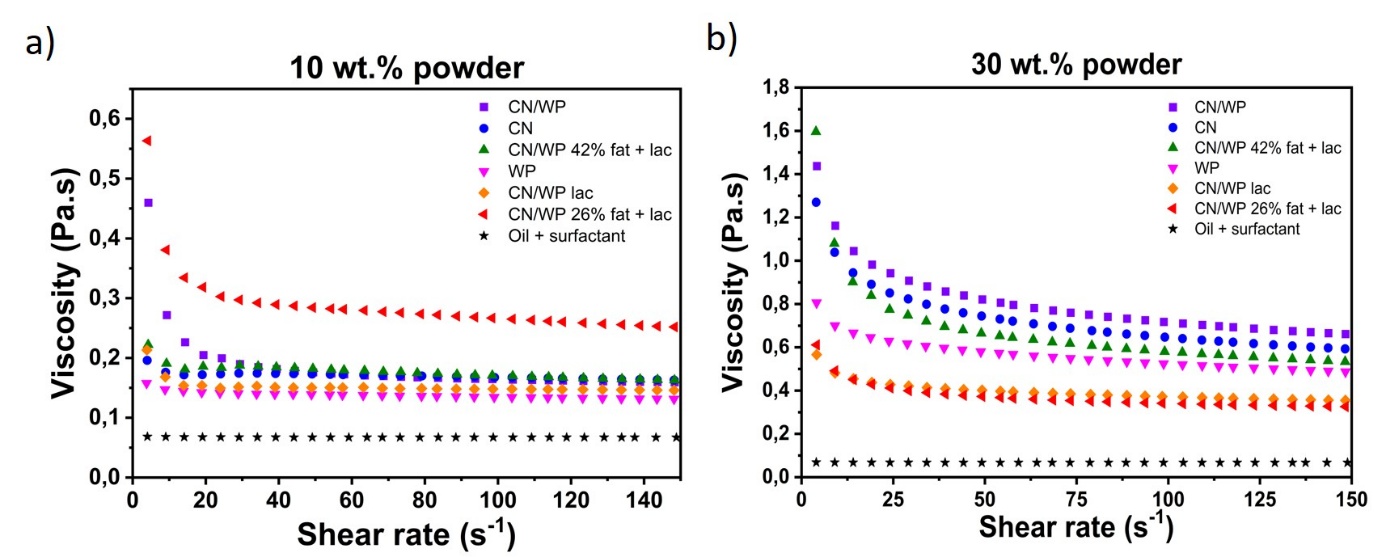


**Figure SI.4:** Viscosity of (a) 10 wt.% and (b) 30 wt.% powder in sunflower oil containing 10 wt.% surfactant: CN/WP (purple square), CN (blue circle), CN/WP 42% fat + lac (green up triangle), WP (pink down triangles), CN/WP lac (orange diamond), CN/WP 42% fat + lac (red left triangle) and sunflower oil containing 10 wt.% surfactant (grey star).


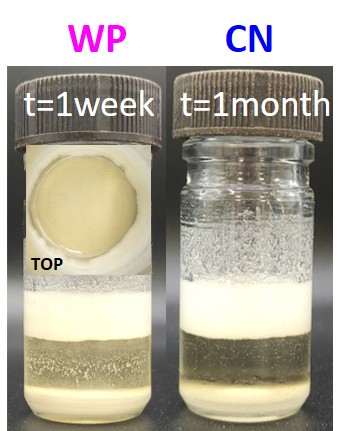


**Figure SI.5**: Photographs of oil foams with the corresponding storage time at room temperature for the same amount of protein (6.10 wt.%): whey protein (first column) and casein (second column). An additional photograph was taken from the top of the foam to show the absence of foam for WP (TOP).


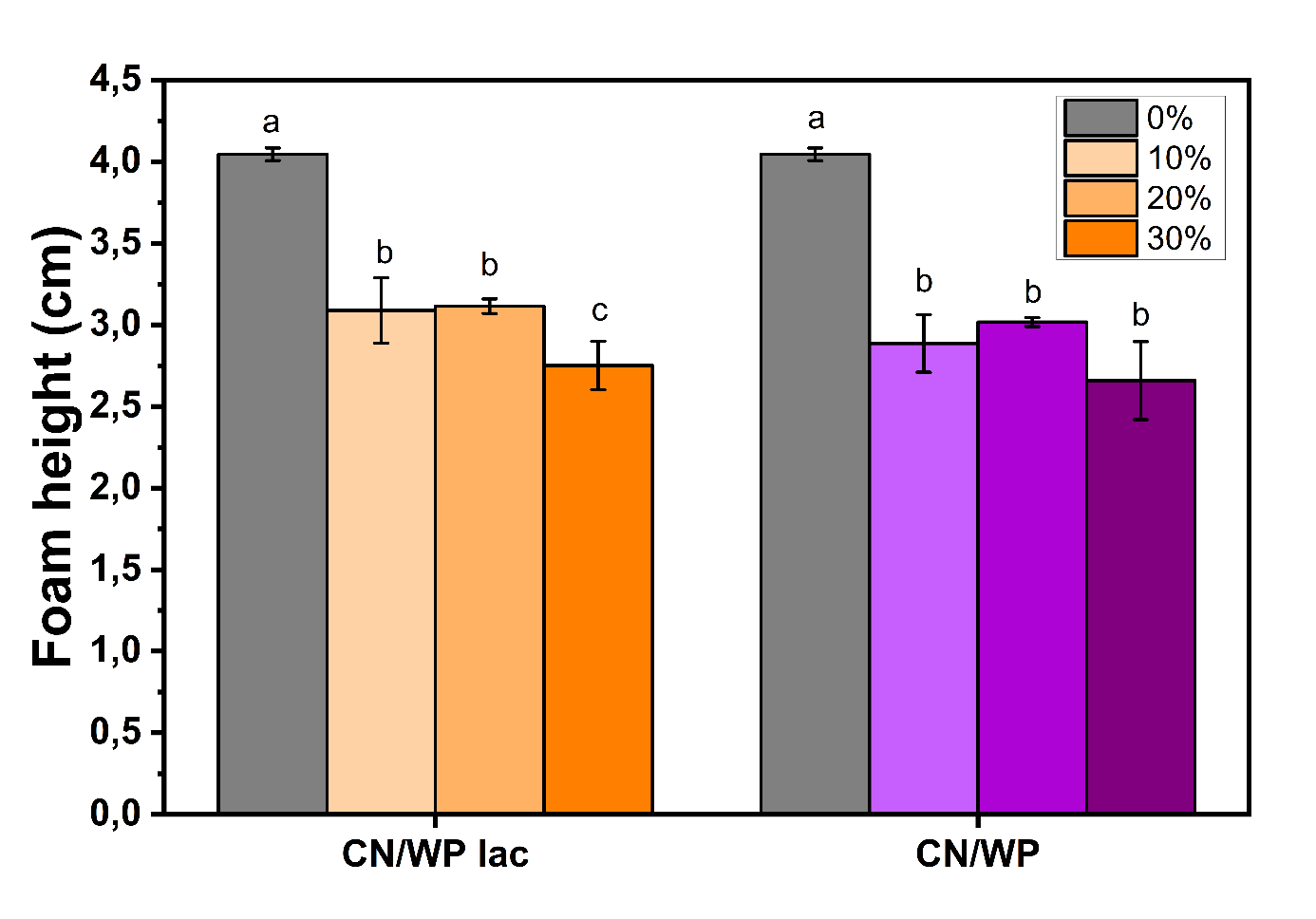


**Figure SI.6**: Foam height obtained just after foaming in centimeters at different concentration from 0 (only surfactants, in grey) to 30 wt.% of dairy powders: CN/WP with lactose (orange), and CN/WP without lactose (purple). The small letters a–c indicates groups of statistical differences according to Tukey’s test (p < 0.05) for each powder as a function of the concentration from 0 to 30 wt.%.


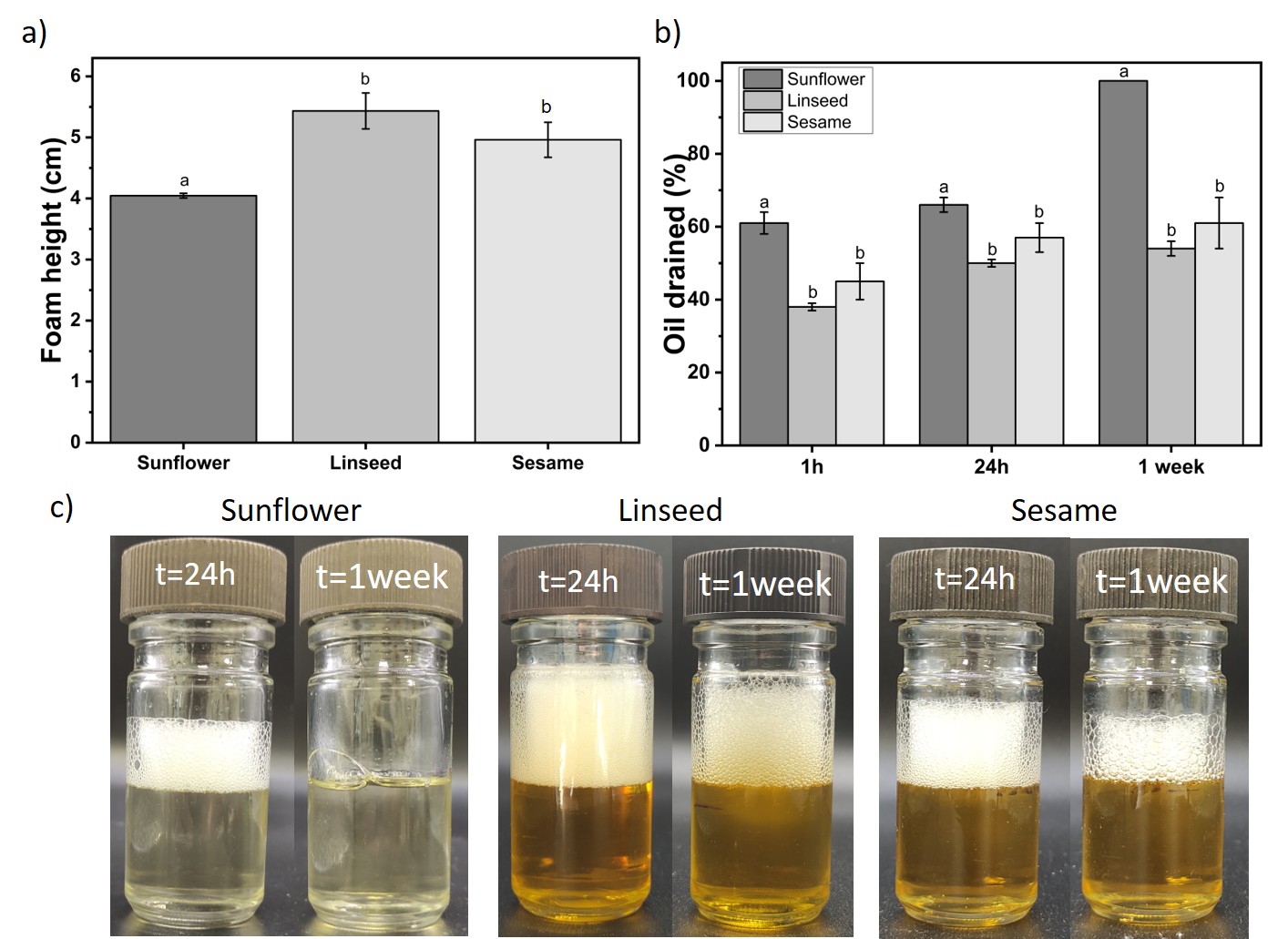


**Figure SI.7:** Oil foams produced with surfactant at 10 wt.%: (a) Evolution of the foam height as a function of the oil nature, (b) Evolution of the oil release (%) with time as a function of the oil nature, (c) Appearance of the oil foams as a function of the oil and time at room temperature. The small letters a–b indicates groups of statistical differences according to Tukey’s test (p < 0.05) for each time as a function of the oil type.


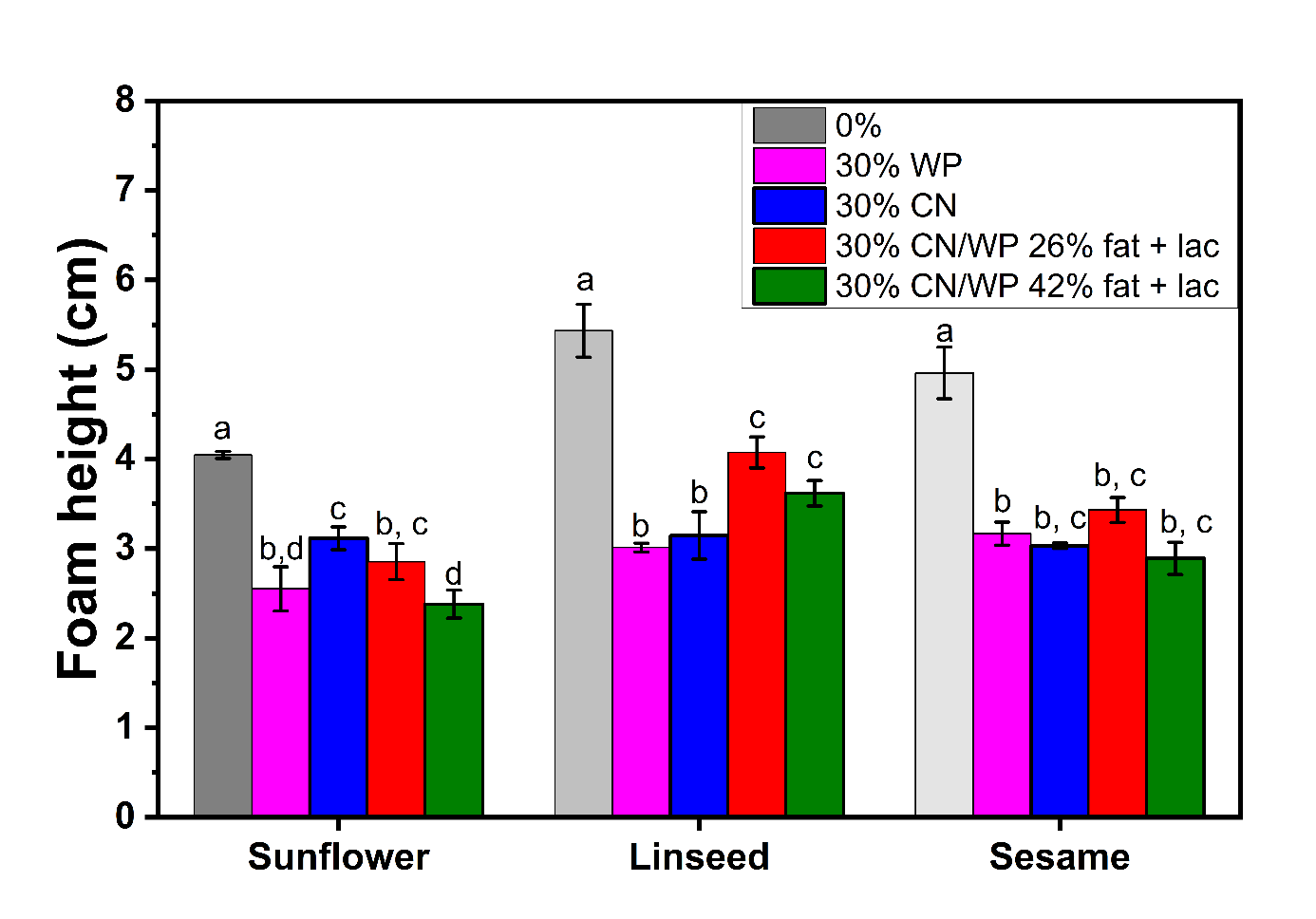


**Figure SI.8:** Oil foams height just after production with different edible vegetable oils with only surfactant at 10 wt.% (grey) and with 30 wt.% of protein powders: WP (pink), CN (blue), CN/WP 26% fat + lac (red) and CN/WP 42% fat + lac (green). The small letters a–d indicates groups of statistical differences according to Tukey’s test (p < 0.05) for each oil nature in function of 30 wt.% of dairy powder nature.


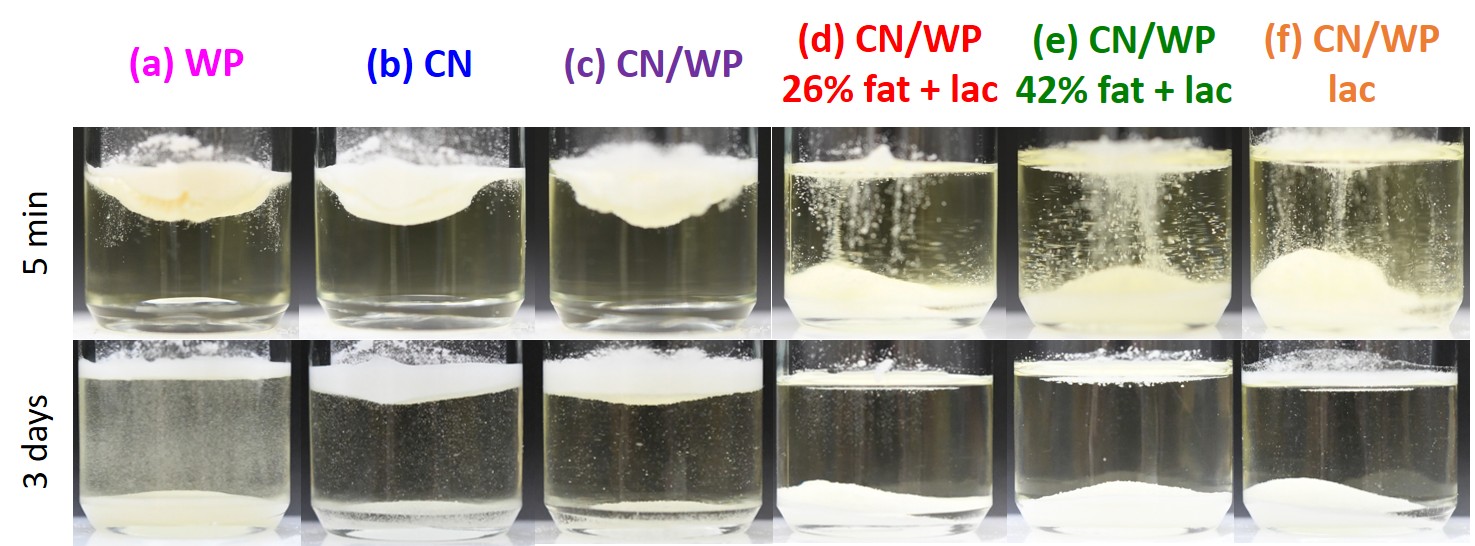


**Figure SI.9:** Pictures at 5 min (first row) and 3 days (second row) after the addition of 0.5 g of the different powders at the air/liquid (pure sunflower oil) surface to evaluate their wetting behavior. All the powders containing milk fat and/or lactose sink rapidly in the liquid (CN/WP 26% fat + lac, CN/WP 42% fat + lac, and CN/WP lac). The milk fat- and lactose-free powders remained at the air/oil surface after 5 min (WP, CN and CN/WP). However, WP sink in the liquid phase after 3 days, whereas CN and CN/WP remained at the air/oil surface.


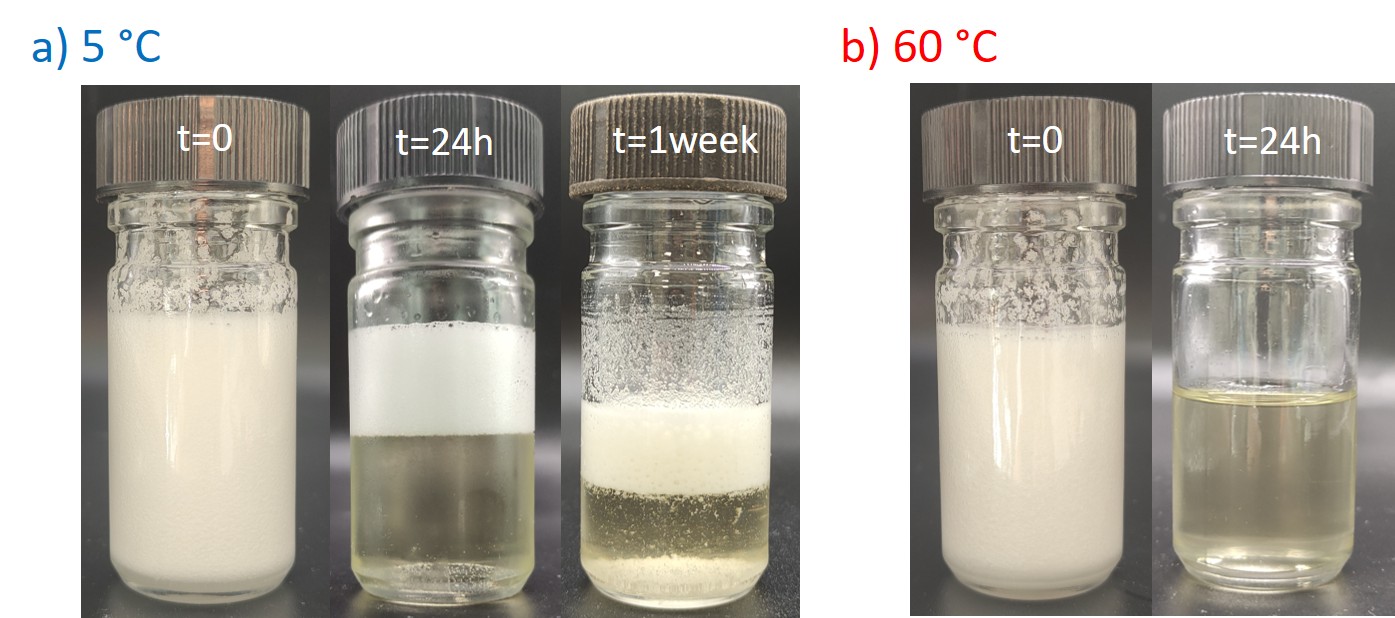


**Figure SI.10:** (a) Photographs of sunflower oil foams based on 10 wt.% surfactant without the addition of protein particles with time at (a) 5 °C and (b) 60 °C.


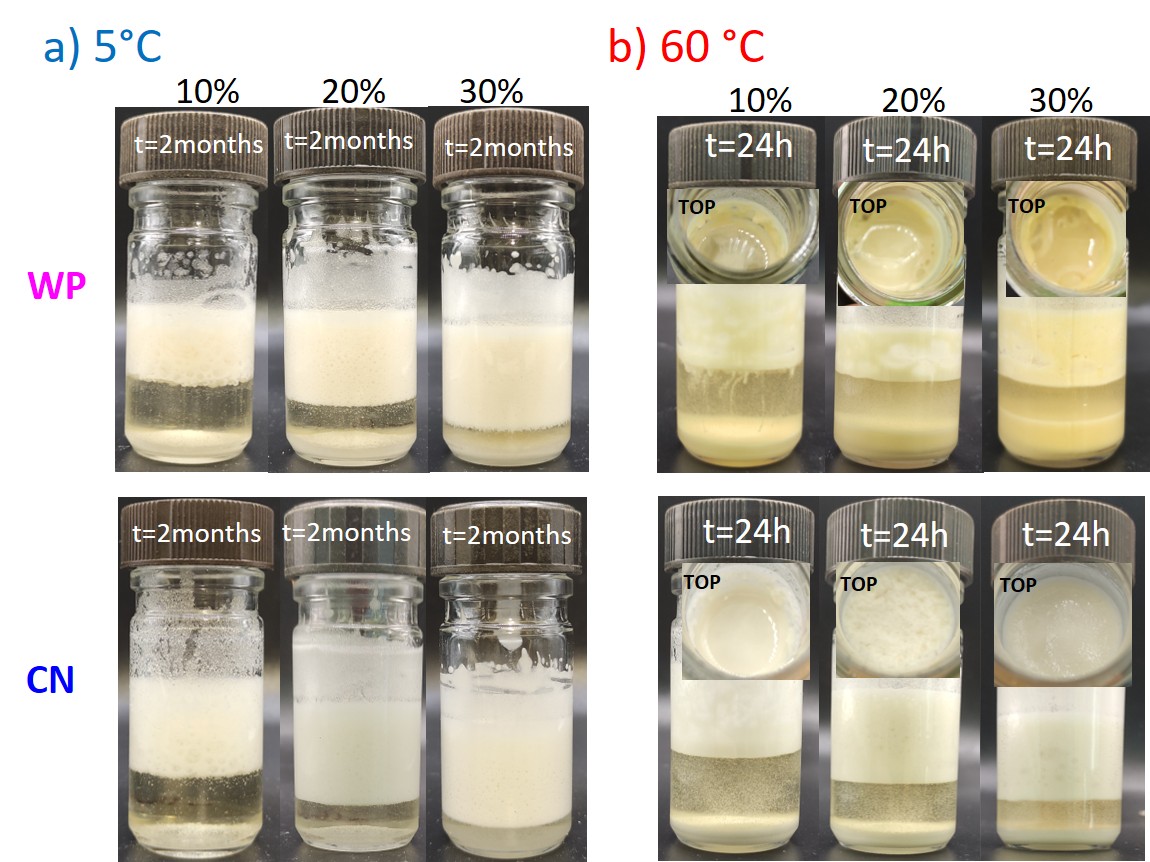


**Figure SI.11:** Photographs of oil foams produced with surfactant at 10 wt.% with either WP (first row) or CN (second row) powder with time at three different powder concentrations (10, 20 and 30 wt.%) and at two different storage temperatures: (a) 5 °C, first column and (b) 60 °C, second column.


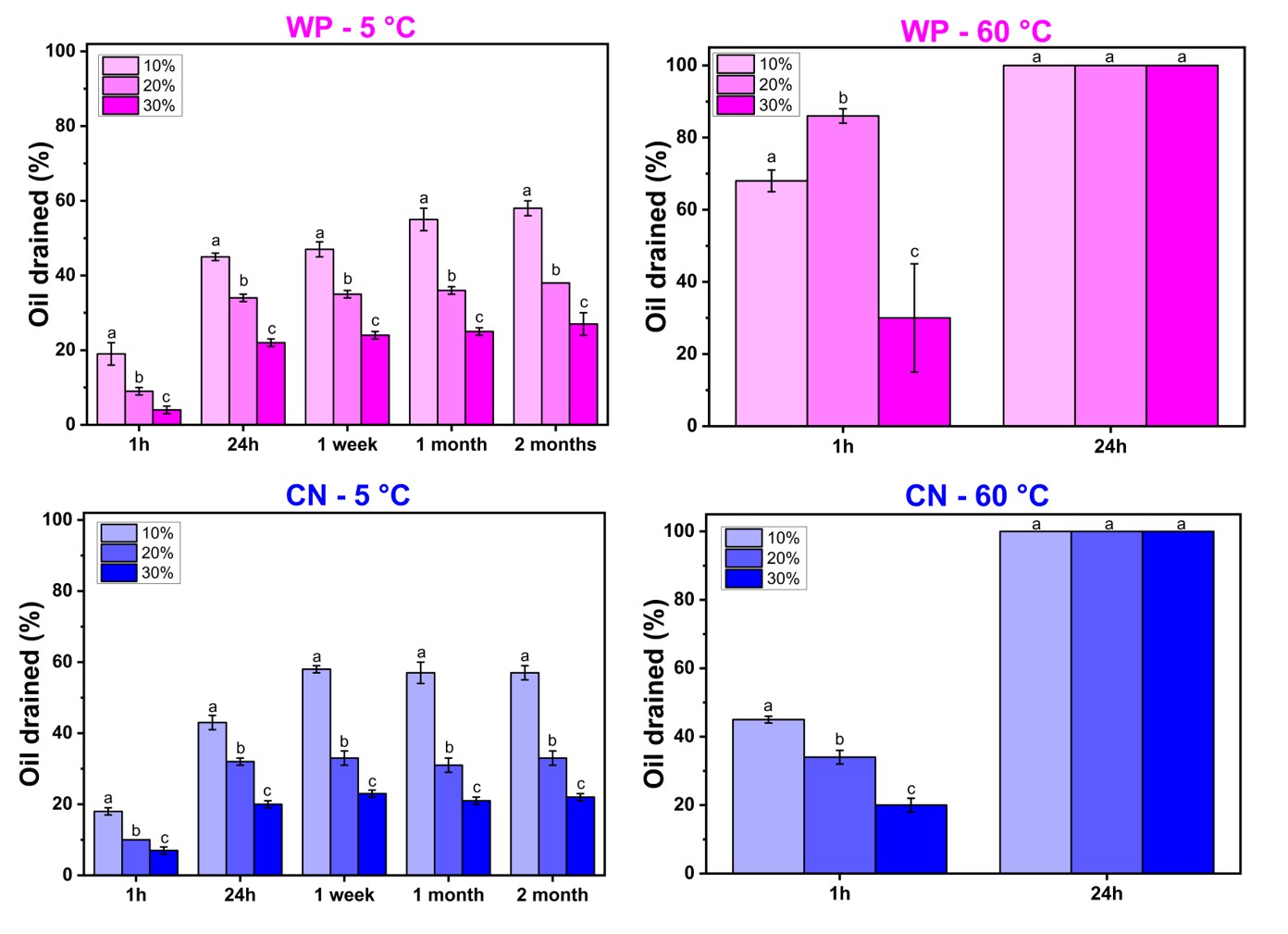


**Figure SI.12:** Percentage of oil drained with time for sunflower oil foams based on 10 wt.% surfactant with the addition of protein particles for the two dairy powders (WP - first row - and CN - second row) at 30 wt.%: 5 °C (first column) and 60 °C (second column). The small letters a–c indicates groups of statistical differences according to Tukey’s test (p < 0.05) for each time as a function of the powder concentration from 10 to 30 wt.%.

**Table SI.1**: Physical characterization of the oil phase without surfactant after protein addition.

|  | WP | CN | CN/WP | CN/WP 26% fat + lac | CN/WP 42% fat + lac | CN/WP lac | Oil |
| --- | --- | --- | --- | --- | --- | --- | --- |
| **Water content in oil phase (%)** | 0.068  ±0.004^a^ | 0.056  ±0.003^b^ | 0.043  ±0.002^c^ | 0.052  ±0.002^b^ | 0.053  ±0.001^b^ | 0.052  ±0.003^b^ | 0.066  ±0.003^a^ |
| **Surface tension (mN/m)** | 37.6±0.9^a^ | 37.7±1.0 ^a^ | 37.4±0.2 ^a^ | 37.4±0.9 ^a^ | 37.2±0.3 ^a^ | 37.6±0.4 ^a^ | 36.8±0.3 ^a^ |

^a-c^ Different letters in each line indicate statistically different means (ANOVA or Tukey’s test, p < 0.05)

**Table SI.2**: Protein content (wt.%) as a function of the amount of dairy powder used (10–30 wt.%), depending on the type of dairy powder.

| **wt.% powder used** | **wt.% of protein** | | | |
| --- | --- | --- | --- | --- |
|  | WP | CN | CN/WP 26% fat + lac | CN/WP 42% fat + lac |
| 10 | 8.78 | 8.52 | 2.69 | 2.03 |
| 20 | 17.55 | 17.03 | 5.38 | 4.07 |
| 30 | 26.33 | 25.55 | 8.06 | 6.10 |

**SI.Note 1:** **Oil foam stability with temperature**

One of the major challenges in foam formulation is maintaining stability across a wide range of storage temperatures. To assess thermal stability, the two best-performing powders, WP and CN, were selected. In addition to their stability at 20 °C already discussed, their stability was also evaluated at lower (5 °C) temperature. At 5 °C, the oil foam without protein was stable only for approximately one week, which equaled its stability at 20 °C, (Figure SI.3a and SI.10a). The stability increased to at least two months when WP or CN powders were present (Figure SI.11 and SI.12). Moreover, protein sedimentation was reduced at 5 °C, suggesting that low temperatures slow down particle sedimentation. Overall, both WP- and CN-stabilized foams exhibited robust long-term stability under refrigerated conditions, with CN consistently yielding slightly superior performance.

In contrast, at 60 °C, rapid destabilization was observed for all oil foams (Figure SI.11 and Figure SI.12). Within few hours, visible sedimentation of WP and CN occurred, and after 24 h, foams had fully collapsed, leaving separated oil and sedimented powder. This indicates that the proteins did not provide any stabilization at high temperatures. Elevated temperatures likely increased the mobility of the particles, accelerated drainage, and weakened bubble stabilization, ultimately leading to foam breakdown.
